# Understanding the relationship between norovirus diversity and immunity

**DOI:** 10.1080/19490976.2021.1900994

**Published:** 2021-03-30

**Authors:** Lauren A. Ford-Siltz, Kentaro Tohma, Gabriel I. Parra

**Affiliations:** Division of Viral Products, Center for Biologics Evaluation and Research, Food and Drug Administration, Silver Spring, Maryland, United States

**Keywords:** Norovirus, gastroenteritis, immunity, vaccines, correlates of protection

## Abstract

Human noroviruses are the most common viral cause of acute gastroenteritis worldwide. Currently, there are no approved vaccines or specific therapeutics to treat the disease. Some obstacles delaying the development of a norovirus vaccine are: (i) the extreme diversity presented by noroviruses; (ii) our incomplete understanding of immunity to noroviruses; and (iii) the lack of a robust cell culture system or animal model for human noroviruses. Recent advances in *in vitro* cultivation of norovirus, novel approaches applied to viral genomics and immunity, and completion of vaccine trials and birth cohort studies have provided new information toward a better understanding of norovirus immunity. Here, we will discuss the complex relationship between norovirus diversity and correlates of protection for human noroviruses, and how this information could be used to guide the development of cross-protective vaccines.

## Introduction

**Figure 1.**
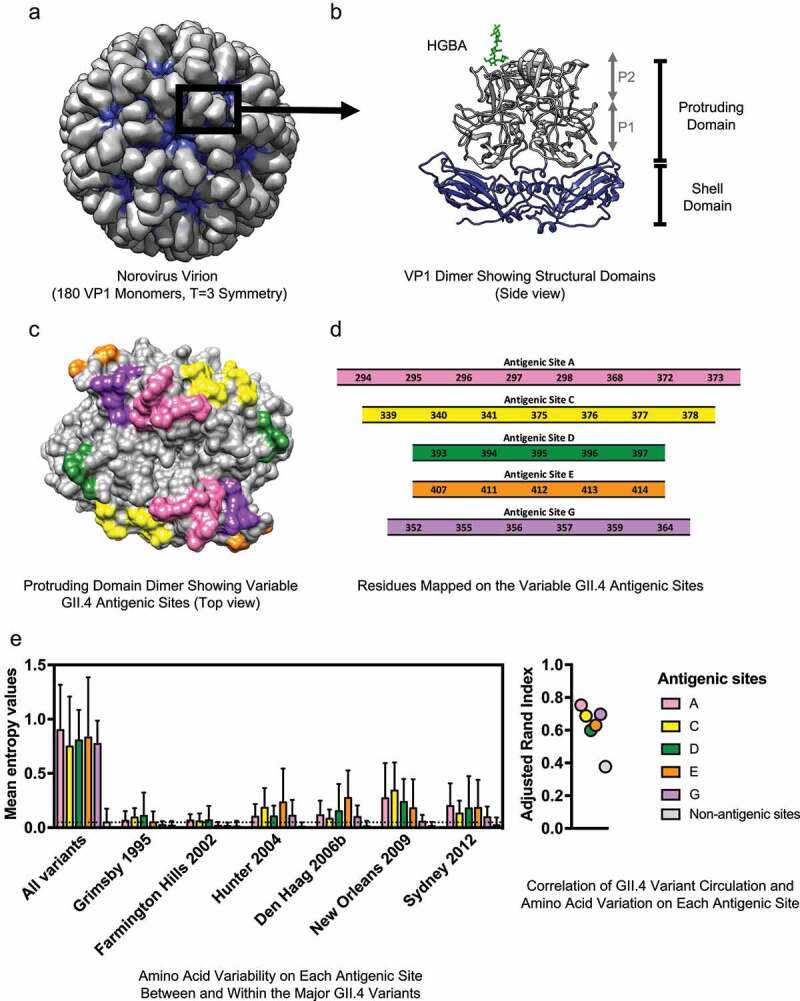
Structure and variable antigenic sites of the human norovirus major capsid protein, VP1. (a) The norovirus capsid is composed of 90 dimers of VP1 arranged in a T = 3 icosahedral symmetry. (b) The VP1 protein is divided into the conserved Shell (S) and the variable Protruding (P) domains. The P domain is further divided into the P1 and P2 subdomains. The surface-exposed P2 subdomain is thought to dictate binding to the cellular attachment factors, histo-blood group antigen (HGBA) carbohydrates (highlighted in green), while the S domain forms the core of the viral particle.^2^ (c) The known variable antigenic sites (A, C, D, E, and G) located on the surface of the P2 subdomain of GII.4 norovirus are highlighted. The structural models were rendered using UCSF Chimera (version 1.11.2) and the following Protein Data Bank (PDB) files: 1IHM and 2ZLE (Norwalk virus, GI.1) and 2OBS (VA387 virus, GII.4 Grimsby variant). (d) Residues mapping on the variable antigenic sites of GII.4 noroviruses. Changes on these sites correlate with the emergence of GII.4 variants. (e) Amino acid variation in the P domain of the VP1 protein and its correlation with GII.4 variant distribution was quantified with Shannon entropy (left) and adjusted Rand index (right). Dataset includes sequences collected from 1995 to 2016, as described in Tohma et al., 2019.^70^ Entropy values were calculated using the Shannon Entropy-One tool, as implemented in Los Alamos National Laboratory (www.hiv.lanl.gov) for six major GII.4 variants. The boxplot shows mean and standard deviation from each antigenic and non-antigenic site of major variants and all-in sequences that included a maximum of 50 randomly subsampled strain/variant to reduce sampling bias (n = 474). The dotted line indicates the mean of entropy values from non-antigenic sites in the subsampled all-in dataset. Adjusted Rand index, in which higher index values indicate a higher degree of correlation between variant distribution and the amino acid variation, was calculated with the subsampled all-in dataset using R and presented in a dot plot. Each circle represents the index from each antigenic and non-antigenic site

Noroviruses are small, icosahedral, non-enveloped, positive-sense, single-stranded RNA viruses that infect different mammal species. The human norovirus genome is ~7.5 kb in length and is divided into three open reading frames (ORF1-3). Upon entry into susceptible cells, the ORF1 is immediately translated as a polyprotein that is processed co- and post-translationally by the viral protease to yield six nonstructural (NS) proteins required for viral replication: NS1/2 (N-term), NS3 (helicase), NS4 (3A-like), NS5 (VPg), NS6 (protease), and NS7 (RNA-dependent RNA polymerase, RdRp). ORF2, which encodes the major capsid protein VP1, and ORF3, which encodes the minor capsid protein VP2, are translated from the subgenomic RNA. The viral capsid presents a T = 3 icosahedral structure consisting of 90 VP1 dimers, and an undetermined quantity of VP2 (Figure 1, panel A).^1,2^ Heterologous expression of VP1 leads to the self-assembly of virus-like particles (VLPs), which are structurally- and antigenically similar to the native virion, although viruses and VLPs with different sizes and symmetries have also been described.^3–5^ In the absence of a robust cell culture system for viral cultivation, VLPs have been instrumental in vaccine research.

Upon infection, the VP1 protein is the major target of B-cell-mediated immune responses, and thus is the primary focus of vaccine development.^6,7^ The VP1 protein is structurally divided into the Shell (S) domain and the Protruding (P) domain (Figure 1, panel B).^2^ Since the S domain is highly conserved across different genotypes, most antigenic sites mapping on this domain are cross-reactive.^8,9^ Unfortunately, most data indicate that protective antibodies map to the variable P domain.^10–14^ The P domain is further divided into the P1 and P2 subdomains. The P1 subdomain is partially conserved, while the P2 subdomain contains the highest sequence variability, including highly variable motifs that are involved in cellular attachment and antibody recognition (Figure 1, panel B).^2^ Both cross-reactive and neutralizing antibodies have been mapped to the P1 subdomain,^12,15^ opening opportunities to induce cross-protective responses.

Based on the genetic diversity of VP1, noroviruses are divided into 10 genogroups and almost 50 genotypes.^16^ Human noroviruses are classified into at least five genogroups that differ by around 40–60% in their amino acid sequences. Genogroups are further subdivided into genotypes that vary between 20% and 40% within each genogroup.^17^ Although humans can be infected by different noroviruses (>30 genotypes, mainly from Genogroups I and II), viruses from the GII.4 genotype are the major cause of large epidemics worldwide.^18,19^

Human noroviruses are the leading cause of viral gastroenteritis in the modern world, and are implicated in upwards of 200,000 deaths worldwide, primarily in children from developing countries.^20,21^ In healthy individuals, norovirus causes acute gastroenteritis (diarrhea and vomiting) that resolves within 24–48 hours, with virus shedding typically lasting between 2 and 8 weeks in the stool.^22^ However, in vulnerable populations (like the elderly, malnourished children, or immunocompromised individuals), the length and severity of disease is increased. Specifically, in immunocompromised individuals, gastroenteritis symptoms and viral shedding can last months or years.^23^ In addition to the disease burden, norovirus presents a major impact on the global economy, with around 4.2 USD billion in direct health-care costs and an additional 60.3 USD billion in indirect costs, i.e. loss of productivity due to absenteeism of work or morbidity.^24^

Based on the natural history of the disease, the general consensus is that a vaccine is the most effective option to mitigate the burden of norovirus disease. Although delayed by major obstacles (i.e. genetic diversity, lack of understanding of immunity, and the absence of a robust cell culture system and animal models), steady progress has been made toward the development of a norovirus vaccine. In this review, we will examine the current knowledge on correlates of protection for human norovirus from the perspective of viral genetic and antigenic diversity, and how this information can be used to design potential cross-protective vaccines.

## The correlates of protection for human noroviruses

A combination of both host genetic factors and immunological responses likely influences norovirus susceptibility, replication, and protection. One of the genetic factors contributing to norovirus susceptibility is the secretor status, which is associated with the presence of histo-blood group antigens (HBGAs) on the surface of epithelial cells. Although a definitive cellular receptor has not yet been identified for human noroviruses, HBGAs have been demonstrated to bind to the VP1 protein and facilitate attachment and/or entry into the cell (Figure 1, panel B).^25–27^

HBGAs are carbohydrates expressed on the surface of most epithelial cells and are the determinants of the ABO and Lewis blood group systems.^28^ Biosynthesis of the different HBGAs is dependent upon multiple glycosyltransferase enzymes, which are responsible for the stepwise addition of monosaccharides onto a precursor carbohydrate molecule. Two of these glycosyltransferases, the fucosyltransferase 1 and 2 (FUT1 and FUT2) enzymes catalyze the addition of a fucose moiety onto the disaccharide precursor via α1,2 linkage, producing the H antigen, which can be further modified.^29^ Mutations that inactivate the *FUT2* gene result in the secretor negative phenotype in humans, which has been shown to provide resistance to infection by certain norovirus genotypes.^26,30–33^ Recently, stem-cell derived human intestinal enteroids (HIE) have been shown to be susceptible to some human norovirus strains.^34^ Using HIE, it has been shown that the expression of a functional FUT2 enzyme in the HIE was necessary for attachment and infection of GI.1 and some GII genotypes,^27^ confirming the relevance of genetic factors to norovirus susceptibility.

Due to the inherent ability of certain norovirus genotypes to bind to HBGA carbohydrates, a carbohydrate blockade assay has been developed in lieu of a cell culture-based neutralization assay. The carbohydrate blockade assay measures the ability of antibodies to block the binding of norovirus VLPs to the HBGA carbohydrates. Several studies have shown that high blockade antibody titers are correlated with disease protection.^35,36^ Importantly, it has been recently shown that carbohydrate blockade assays and neutralization in HIE present similar patterns in antibody reactivity.^11,37^ As the HIE culture system exhibits evident disadvantages, such as limited scalability to high-throughput and/or the inability of certain noroviruses to replicate in the cells, the blockade assays are a good alternative to determining the antigenic relationships between strains and to study antibody-mediated protection.^11,12,37,38^

While some animal models have been developed for human noroviruses,^14,39–41^ they either do not recapitulate disease or replication has been shown for only a limited number of strains. Thus, human challenge studies have facilitated the study of norovirus immunity. Early studies have shown short-term protection (4–14 weeks post initial challenge) against the homologous virus,^42,43^ and the lack of cross-protection between heterologous viruses, today identified as GI and GII viruses.^44^ Protection from disease was associated with high levels of norovirus-specific antibodies before infection.^42,43,45,46^ A caveat of the early studies is that the viruses used for challenge were not titered,^43^ so an overwhelming infectious dose, which does not represent natural conditions, could have masked the effect of immunity mounted by previous infections.^47^

Challenge studies and analyses of natural infections have revealed that both serum and mucosal antibodies are involved in the immune response. An increase in norovirus-specific IgG and IgA has been reported after infection,^48^ with higher titers of preexisting salivary IgA being correlated with protection.^33,45^ Interestingly, when compared to norovirus-specific IgG antibodies, a higher proportion of IgA antibodies blocked the binding of norovirus to carbohydrates and exhibited a more potent blockade titer.^49^ IgAs were also shown to be superior to IgG in terms of murine norovirus (MNV) neutralization *in vitro* and protective function *in vivo*.^50^ Simlarly, a higher percentage of serum IgA antibodies bound to the P domain, which contains neutralizing epitopes, compared to serum IgG, which equally recognized the P domain and the conserved S domain.^50^ Additional studies are warranted to determine the role of IgA in virus neutralization. In addition to serum and salivary IgA antibodies, fecal IgA also are induced after infection.^33,45,51^ Although preexisting levels of norovirus-specific fecal IgA were not correlated with protection, in individuals who developed gastroenteritis, fecal IgA levels were inversely correlated with peak viral load in the stool,^45^ suggesting a role in the control of viral replication. These studies underline both serum and mucosal immunity as major players in the immune response against norovirus.

## The impact of norovirus diversity on immunity

Although the genetic diversity of human noroviruses is well established, the antigenic diversity and its role in immunity, especially between genotypes, is less understood. A study that analyzed the cross-reactivity of hyperimmune sera produced from 26 norovirus VLPs, representing around 18 genotypes, showed a variable range of cross-reactivity between the different genotypes via ELISA.^52^ However, the observed cross-reactivity is likely influenced by non-neutralizing antibodies targeting conserved regions of the VLPs, and thus may not truly represent the functional antigenic relationships between the different genotypes. Preliminary neutralization experiments in HIE and analyses of natural reinfection cases over time suggest that some norovirus genotypes are antigenically distinct from each other.^11,17,42,53–55^ Despite all these studies, extensive analyses of the functional (blockade/neutralizing) antibodies between all human norovirus genotypes have not been performed.

Another confounding factor is that contrasting evidence on the duration of immunity has been documented. While challenge studies suggest a short duration of protection,^42,43^ mathematical modeling based on norovirus community transmission suggests that immunity may last up to 9 years.^56^ Moreover, studies have shown that individuals can be infected with multiple genotypes over the course of their lifetimes.^42,51,53–55,57,58^ Whether these reinfection patterns are associated with short duration of immunity, genotype antigenic differences, biological properties of emerging viruses, or all of the above have not yet been fully determined.

Based on reinfection patterns recorded in longitudinal and birth cohort studies, and anecdotical reports, human noroviruses were recently clustered into genetically similar groups called “immunotypes,” where infection with a genotype precluded disease with genotypes within the same immunotype.^17^ This symptomatic reinfection pattern suggests that multiple exposures over a lifetime could provide cross-protective immunity. Indeed, there are data supporting that the history of infection also influences the magnitude and breadth of the immune response. A rapid anamnestic response specific to the initial infecting genotype has been recorded in children and adults.^42,51^ Moreover, serum IgG and IgA collected from adults infected with norovirus were able to block the binding of VLPs from multiple genotypes to carbohydrates^59,60^ and immunization with norovirus VLP-based vaccine candidates induces antibodies against heterologous strains,^61,62^ probably by recalling antibodies specific to prior infections.^61^ Whether cross-protective antibodies would develop in naïve individuals upon vaccination remains to be determined. Thus, there appears to be a multifaceted relationship between previous infections and cross-protective immune responses, and further studies are warranted to determine whether these genetically related viruses share similar antigenic properties that could result in enhanced cross-protection.

One exception to this pattern of reinfection was repeated infections with GII.4 viruses, the genotype responsible for the majority of cases in humans.^18,19^ GII.4 reinfections are likely due to the ever-changing nature of this genotype, which results in antigenically distinct variants emerging to cause outbreaks around the world every 2–8 years.^18,19^ In contrast, non-GII.4 noroviruses appear to be “static” in nature, such that strains that are detected decades apart remain similar at the amino acid level.^17^ Although certain non-GII.4 noroviruses (GI.3, GII.1, GII.2, GII.6, GII.17, among others) can be subdivided into variants, individual viruses within each variant show limited diversification at the amino acid level.^63^ Thus, the evolving nature of GII.4 noroviruses needs to be especially considered in vaccine design.

The VP1 antigenic topology of GII.4 norovirus has been extensively characterized. Bioinformatics and experimental analyses (i.e. comparing blockade responses of human sera collected from GII.4 outbreaks or hyperimmune sera produced in experimental animals against VLPs of representative GII.4 variants^64,65^) conducted over the past decade have identified five variable antigenic sites that are involved in the chronological emergence of new variants. The precise residues on VP1 involved in each of those variable antigenic sites have been mapped using human and animal monoclonal antibodies (mAbs) (Figure 1, panel C). Thus, antigenic site A consists of residues 294–298, 368, 372, 373; antigenic site C of 339–341, 375–378; antigenic site D of 393–397; antigenic site E of 407, 411–414; and antigenic site G of 352, 355–357, 359, 364 (Figure 1, panel D).^66–70^ Most of these sites are on the loops and/or outer surface of the P2 subdomain where they can interact with neutralizing antibodies and attachment factors. Notably, although the overall structure of the capsid is mostly conserved between the different variants of GII.4, the length varies among genotypes. Thus, careful interpretation of these data should be considered when extrapolating GII.4 antigenic sites to other genotypes.

While the antigenic sites present low levels of intra-variant diversification, most amino acid changes occur at the inter-variant level, suggesting that changes on these residues are important in differentiating the GII.4 variants (Figure 1, panel E, left). Changes on these antigenic sites, particularly antigenic sites A, C, and G, also correlate with the circulation patterns of GII.4 variants (Figure 1, panel E, right).^70^ Notably, while mutations in single antigenic sites can abolish mAbs binding,^69,70^ large-scale antigenic studies suggest that multiple sites are required for antigenic diversification of GII.4.^38^ Indeed, structural analyses identified mAbs with footprint spanning multiple antigenic sites, suggesting that the virus may need to change multiple sites to evade the host immune response.^13,68,71^

The identification and manipulation of immunodominant epitopes are important factors in designing a vaccine that is able to induce functional, protective antibodies against a foreign antigen.^72^ It was demonstrated that the emergence of the GII.4 New Orleans/2009 variant was associated with changes in the immunodominant antigenic site A, whereby ~40% of blockade antibodies from human outbreak sera were directed against this site.^69^ Similarly, using polyclonal guinea pig sera, our group showed that antigenic sites A and G from the GII.4 Sydney/2012 variant were both important immunodominant blockade sites.^70^ Whether these immunodominant sites can induce cross-protective antibodies against multiple strains or if these antibodies are variant-specific remains to be determined. For example, as antigenic site G is more conserved compared to the other major antigenic sites/motifs, antibodies targeting site G could potentially provide protection against multiple GII.4 variants. These immunodominant sites may potentially mask protective epitopes from the immune response as seen in other viruses (e.g. influenza and HIV).^73–75^

Some studies have predicted additional epitopes that are dependent upon capsid conformation and assembly. Noroviruses, among other viruses, are thought to be dynamic in their structure, i.e. the capsid exists in multiple states. This phenomenon is termed “viral breathing” and is thought to regulate antibody access to epitopes,^76^ such as the conserved antigenic site F (residues 327 and 404).^77,78^ In addition, although initial structural studies have determined that the norovirus capsid arranges with a T = 3 icosahedral symmetry, GII.4 norovirus can also assemble into larger particles with T = 4 icosahedral symmetry. These T = 4 particles are made up of 240 copies of VP1 that adopt different conformations that may present unique epitopes.^4,5^ Interestingly, the formation of T = 4 particles seems restricted to VLPs produced with the baculovirus expression system, as images of native GII.4 virions reveal the presence of particles of the expected size (~30 nm), as well as smaller particles (~18 nm) that are likely T = 1.^34^ Thus, the role of VLPs with T = 4 icosahedral symmetry for vaccine design should be further explored.

In contrast to GII.4, the evolution and antigenicity of non-GII.4 noroviruses are less understood. Our group recently showed that hyperimmune guinea pig sera produced against various norovirus genotypes exhibited specific blockade and neutralizing titers against homologous genotypes but not against heterologous genotypes,^11^ suggesting that a cross-protective vaccine would likely need multiple components. Vaccine design may also be complicated by the fact that, similar to GII.4, certain non-GII.4 genotypes (GII.17, GII.6, etc.) can be subdivided into variants.^17^ Furthermore, although GII.4 is responsible for the majority of outbreaks, non-GII.4 noroviruses can also emerge and predominant in the population. In the winter season of 2014–2015, GII.17 unexpectedly overtook GII.4 to become the predominant genotype in several parts of East Asia.^79,80^ The epidemic GII.17 noroviruses were shown to be antigenically distinct from previous strains via carbohydrate blockade assays using human and hyperimmune mouse sera.^81^ Similarly, GII.2 emerged and predominated in several countries in the winter of 2016–2017.^82–84^ Thus, an ideal vaccine would need to protect against multiple genotypes.

Unlike GII.4 noroviruses, the antigenic sites of non-GII.4 genotypes have not been well defined. Exchanging the amino acid residues 393–396 from the GII.17/2015 outbreak strain to an archival GII.17/1978 strain altered the blockade activity of monoclonal and polyclonal antibodies, suggesting that these residues may be involved in defining the antigenicity of these GII.17 strains.^85^ In contrast, the antigenicity of GII.2 variants across decades (1976–2010) appears to be similar, as sera collected from humans challenged with GII.2 SMV/1976 were able to block contemporary GII.2 VLPs, although to a lesser extent compared to the homotypic VLP.^86^ Interestingly, mAbs produced against GII.2 SMV/1976 exhibited blockade titers against a panel of GII.2 strains spanning decades.^86^ Thus, certain genotypes (e.g. GII.2) appear to be more stable in their antigenic evolution over time.

## Current and prospective vaccines

As previously discussed, the extreme diversity of human noroviruses is one of the major hurdles in the development of cross-protective vaccines. Based on data gathered from challenge studies,^14,44^ current norovirus vaccine candidates are bivalent in formulation, consisting of a GI and a GII component. Two candidates are currently in clinical trials: (i) a VLP-based vaccine administered via the intramuscular route (developed by Takeda Pharmaceuticals) and (ii) an oral vaccine adenovirus vector expressing the VP1 gene (developed by Vaxart, Inc.). Takeda’s VLP-based vaccine candidate was well tolerated in healthy volunteers and induced humoral and mucosal immunity against both antigens, a GI.1 VLP and a consensus GII.4 VLP derived from three different GII.4 variants.^62,87^ Vaxart recently announced the results of a Phase 1b clinical trial to test the safety and immunogenicity of the vector-based, bivalent (GI.1 + GII.4) vaccine formulation.^88^ The bivalent vaccine was well tolerated and induced robust IgA responses in the majority of vaccinees with no observed interference. Vaccination with monovalent vectors expressing GI.1 or GII.4 VP1 were also well tolerated and resulted in an increase in antibody titers against the antigens.^88,89^

In addition to the vaccine candidates in clinical trials, several investigational vaccines are currently being tested in preclinical studies. Many of these clinical and pre-clinical vaccine candidates have been reviewed (see ref.^47^) and are summarized in Table 1. Briefly, a trivalent combination vaccine consisting of a GI.3 VLP, a GII.4 VLP, and a recombinant VP6 protein from rotavirus induced moderate levels of serum IgG and blockade antibodies in immunized mice against homologous and heterologous norovirus and rotavirus strains.^90,91^ The foundation of this particular vaccine is the recombinant VP6 rotavirus protein, which forms a tubular-like capsule and acts as an adjuvant to the norovirus VLPs.^91^ Although norovirus VLPs are often produced using the baculovirus expression system,^62,92^ expression of the VP1 protein in a plant-based tobacco mosaic virus system also results in production of immunogenic VLPs.^93^ A second adenovirus-vectored vaccine candidate expressing the VP1 of a single GII.4 strain induced mucosal immunity and a balanced cellular Th1/Th2 response in experimental animals.^94^ In addition to adenovirus, Vesicular Stomatitis virus (VSV) is also used as a vector for expression of norovirus VP1. Immunization of mice with a single dose of recombinant (r) VSV-VP1 resulted in strong serum and mucosal responses, as well as the induction of norovirus-specific T-cells in some animals.^95^ Finally, another vaccination strategy relies on the singular expression of the P domain of VP1; this results in the formation of highly immunogenic *P*-particles that are antigenically similar to whole VLPs and that can be used as an alternative to VLP vaccination.^96^ A study on GII.4 P particles showed that hyperimmune sera in immunized animals was able to block the binding of the GII.4 VLP to carbohydrates, suggesting that the antibodies have potentially neutralizing activity.^97^ Thus, many potential vaccine candidates utilizing a variety of approaches for human norovirus are being explored. However, it remains to be seen whether the vaccines induce broad protection against the other genotypes, some of which recently have emerged to cause large outbreaks in different countries (i.e. GII.17, GII.2) or in vulnerable populations (i.e. children, the elderly).

**Table 1.** Current and prospective human norovirus vaccines

Vaccine Candidate	Affiliation/Investigators	Current Stage	Antigen	Adjuvant (if any)	Route of Administration	Citation
VLP-based, bivalent	Takeda Pharmaceuticals	Phase 2b	GI.1, GII.4c*	Monophosphoryl Lipid A (MPL) + Alum	intramuscular	[62], [87]
Recombinant adenovirus vector expressing VP1, bivalent	Vaxart, Inc.	Phase 1b	GI.1, GII.4	dsRNA hairpin (TLR3 agonist)	oral	[88], [89]
Norovirus VLP and recombinant rotavirus combination, trivalent	Tamminen et al.	preclinical	GI.3, GII.4, recombinant rotavirus VP6	rVP6**	intramuscular	[80], [91]
VLP-based, monovalent	Santi et al.	preclinical	GI.1 produced in Tobacco Mosaic virus expression system	Cholera toxin (CT)	oral	[93]
Recombinant adenovirus vector expressing VP1, monovalent	Guo et al.	preclinical	GII.4	n/a	intranasal	[94]
Recombinant vesicular stomatitis virus vector expressing VP1, monovalent	Ma et al.	preclinical	GII.4	n/a	intranasal and oral	[95]
P-particles, monovalent or as a carrier linked to rotavirus VP8	Su et al.; Tan et al.	preclinical	GII.4 P-particle expressed in *E. coli* or yeast, alone or with rotavirus VP8	Freund’s adjuvant	subcutaneous	[96], [97]

*GII.4c = GII.4 consensus VLP derived from three variants ** rVP6 = recombinant rotavirus VP6 protein n/a = not applicable

## Overcoming diversity to develop cross-protective vaccines

One of the goals in vaccine development is the induction of cross-protective antibodies. Studies of the human antibody repertoire following vaccination with the bivalent VLP vaccine candidate revealed three classes of antibodies: (i) antibodies that bound a wide array of genotypes but did not exhibit blockade activity, (ii) antibodies that exhibited binding and blockade activity specifically against the GII.4  consensus VLP and historical GII.4 strains, and (iii) a single mAb – A1431 – that recognized and blocked/neutralized all tested GII.4 strains, including forthcoming variants.^12^ A1431 was mapped to the cleft between the P1 and P2 subdomains of VP1, which consists of amino acids that are highly conserved within the multiple GII.4 variants. As this site did not overlap with the HBGA binding sites, the mechanism of blockade/neutralization is likely due to steric hindrance.^12^ Likewise, mouse mAb 5B18 was mapped to a conserved region of the P1 subdomain located near the P1/Shell interface.^15,98^ Interestingly, the antibody-binding site was buried within the P1 domain and was only transiently exposed to the surface, perhaps due to the “viral breathing” phenomenon or VLP disassembly. Antibodies against such hidden sites may be involved in cross-protective immunity, and further research is warranted to understand how to elicit responses against such hidden epitopes.

Although blockade/neutralizing antibodies are thought to play a major role in protection, the potential role of non-neutralizing, cross-protective antibodies in immunity should not be disregarded. Non-neutralizing IgA antibodies against influenza A virus were recently shown to inhibit the release of multiple influenza A strains from infected cells.^99^ Similarly, administration of cross-reactive, non-neutralizing antibodies protected mice from lethal influenza B virus challenge.^100^ In contrast, IgG1 antibodies against influenza virus were shown to inhibit the cross-protective effect of IgG2 antibodies after vaccination in mice, suggesting that antibody isotypes play a major role in pathogen control.^101^ Other mechanisms by which non-neutralizing antibodies enhance or antagonize the immune response have been reviewed.^102^ Vaccine design should focus on targeting conserved, protective epitopes and inducing the proper Ig subtype, perhaps by manipulating the presentation of the antigen and/or by selecting adjuvants that will enhance the antibody repertoire as shown for influenza and human papilloma virus.^103,104^

In conclusion, the large diversity of human noroviruses presents a problem that must be overcome to develop a cross-protective vaccine. Historically, the lack of a robust cell culture system and suitable small animal model contributed to the difficulties in the study of human norovirus antigenicity and correlates of immune protection. Recently, several advances have been made, including the advent of a cell culture system that is susceptible to certain norovirus strains^34^ and the conclusion of early-phase vaccine trials,^87,89^ helping us to better understand the role of norovirus diversification in immunity. Future research that focuses on the synergy of duration of immunity, genotype antigenic differences, B- and T-cell immunodominance, and acquisition of biological properties that enhance replication and transmission will facilitate the development of effective control strategies against noroviruses.
